# 28 days of creatine nitrate supplementation is apparently safe in healthy individuals

**DOI:** 10.1186/s12970-014-0060-9

**Published:** 2014-12-20

**Authors:** Jordan M Joy, Ryan P Lowery, Paul H Falcone, Matt M Mosman, Roxanne M Vogel, Laura R Carson, Chih-Yin Tai, David Choate, Dylan Kimber, Jacob A Ormes, Jacob M Wilson, Jordan R Moon

**Affiliations:** Department of Health Sciences and Human Performance, The University of Tampa, Tampa, FL USA; MusclePharm Sports Science Institute, MusclePharm Corp., Denver, CO USA; Metropolitan State University, Denver, CO USA; Department of Sports Exercise Science, United States Sports Academy, Daphne, AL USA

**Keywords:** Creatine, Nitrate, Safety, Health, Hematology, Immunity, Immune system

## Abstract

**Background:**

Creatine monohydrate has become a very popular nutritional supplement for its ergogenic effects. The safety of creatine monohydrate has previously been confirmed. However with each novel form of creatine that emerges, its safety must be verified. Therefore, the purpose of this study was to examine the safety of a novel form of creatine, creatine nitrate (CN), over a 28 day period.

**Methods:**

58 young males and females (Pooled: 24.3 ± 3.9 years, 144.9 ± 8.0 cm, 74.2 ± 13.0 kg) participated in this study across two laboratories. Subjects were equally and randomly assigned to consume either 1 g (n = 18) or 2 g (n = 20) of CN or remained unsupplemented (n = 20). Blood draws for full safety panels were conducted by a trained phlebotomist prior to and at the conclusion of the supplementation period.

**Results:**

Pooled data from both laboratories revealed significant group x time interactions for absolute lymphocytes and absolute monocytes (p < 0.05). Analysis of the 1 g treatment revealed lab x time differences for red blood cell distribution width, platelets, absolute monocytes, creatinine, blood urea nitrogen (BUN):creatinine, sodium, protein, and alanine aminotransferase (ALT) (p < 0.05). Analysis of the 2 g treatment revealed lab x time differences for BUN:creatinine and ALT (p < 0.05). BUN and BUN:creatinine increased beyond the clinical reference range for the 2 g treatment of Lab 2, but BUN did not reach statistical significance.

**Conclusion:**

Overall, CN appears to be safe in both 1 g and 2 g servings daily for up to a 28 day period. While those with previously elevated BUN levels may see additional increases resulting in post-supplementation values slightly beyond normal physiological range, these results have minor clinical significance and are not cause for concern. Otherwise, all hematological safety markers remained within normal range, suggesting that CN supplementation has no adverse effects in daily doses up to 2 g over 28 days and may be an alternative to creatine monohydrate supplementation.

**Electronic supplementary material:**

The online version of this article (doi:10.1186/s12970-014-0060-9) contains supplementary material, which is available to authorized users.

## Introduction

In regards to ergogenic supplementation, creatine’s effects have been researched in humans since the early 1990’s. Earnest and colleagues examined supplemental creatine’s effects on both body composition and muscular performance [1]. After supplementing with creatine for only 2 weeks, subjects were tested with three 30s Wingate sprints, 1 repetition maximum in the bench press, and repetitions to failure at 70% 1RM. They discovered that creatine supplementation improved repeated sprint performance, bench press strength, fatigue resistance, body weight, and relative lifting volume. They also observed an insignificant trend for fat free mass. Creatine has since been verified as a potent supplement for each of these and more variables on numerous occasions [2-5].

Creatine supplementation’s ergogenic effects are largely explained by increasing intramuscular phosphocreatine (PCr) stores [6]. The increase in PCr allows adenosine diphosphate (ADP) to be readily rephosphorylated to adenosine triphosphate (ATP) [7,8], which is depleted rapidly during strenuous exercise. Thus, increasing the amount of PCr through creatine supplementation increases the capacity to rephosphorylate ADP to ATP, and this allows the athlete to resist fatigue and maintain a higher level of performance [6,9-11].

This increase in fatigue resistance created by creatine supplementation allows for increased training volume. As demonstrated by several laboratories, creatine supplementation is able to increase repetitions to failure [1,12]. Therefore, many athletes have adopted creatine use during pre-season and/or off-season training for the cumulative effects of increased training volume [13], and they are not using creatine exclusively for acute performance benefits. In addition to chronic performance benefits, creatine aids those seeking to increase muscle mass [2-5,14]. Muscle mass increases can be explained in part by creatine increasing training volume [1], but it is also partially explained by other factors. Saremi et al. [5] confirmed creatine’s effects on increasing lean body mass after chronic resistance training, and in addition, they observed decreased levels of myostatin in the creatine supplemented group. Olsen and colleagues have demonstrated increased myonuclei and muscle fiber area after 16 weeks of concurrent resistance training and creatine supplementation [15]. Additionally, 12 weeks creatine supplementation has been demonstrated to increase myogenic regulatory factor expression and myogenin, both of which initiate transcription and regulate gene expression, which likely contribute to the anabolic effects of creatine [16].

Similarly, dietary nitrate has been receiving more attention for its effects on energy efficiency and work capacity. Nitrate supplementation, typically in the form of foods or juices high in inorganic nitrate such as beet root juice, has primarily been demonstrated to decrease oxygen cost despite maintaining work load [17-21]. This has led to improved performance [22,23] and increased muscle contractile efficiency by decreasing the ATP cost of muscle contraction [17,24]. Despite health benefits of nitrate supplementation [25,26], concerns have been raised over nitrosamines [27,28], and consumers may still be weary of consumption. It is also possible that healthcare practitioners may not fully understand the role of nutritional supplements, nutrition, and exercise, and therefore, they may raise unfounded safety concerns.

Because of the ergogenic effects of creatine and phosphocreatine and the ATP sparing effects of nitrate [17], the two supplements have been combined efficaciously along with other ingredients [29]. While both nitrates and creatine should help enhance performance similar to taking both ingredients alone, little is known about the safety of creatine bound to a nitrate when ingested regularly. Therefore, the purpose of this study was to examine the safety of a novel form of creatine, creatine nitrate (CN). We hypothesized that CN supplementation would not produce abnormal changes in hematological safety markers.

## Methods

### Experimental design

In a randomized design, a total of 58 subjects were recruited for this study across two laboratories. Subjects were randomly divided into control (CRL), 1 serving (G1), or 2 serving (G2) groups. Wherein, the CRL group did not supplement and G1 and G2 consumed 1 serving (1 g) and 2 servings (2 g) of CN (Iron Cre3™, MusclePharm Inc., Denver, CO), respectively, every day for 28 days in an unblinded manner. In addition to the CN, the supplement contained 1 g of carbohydrate, 500 mg of vitamin C, 500 IU of Vitamin E, 18 mg of Calcium, and 800 mg of a proprietary blend consisting of taurine, coconut water powder, and L-glutamine per serving. Supplement containers were weighed prior to and following the supplementation period and supplement consumption logs were completed by each participant to ensure compliance. Blood draws were conducted prior to and at the conclusion of the supplementation period. MusclePharm Sports Science Institute (Lab 1) received approval from the MusclePharm Sports Science Institute IRB, and the University of Tampa Human Performance and Nutrition Laboratory (Lab 2) received approval from the University of Tampa IRB. Each subject was provided written informed consent prior to participation in the study.

### Participants

Forty-two subjects (25.2 ± 4.9 years, 173.8 ± 10.2 cm, 77.2 ± 16.7 kg, CRL n = 20, G1 n = 10, G2 n = 12) were recruited by Lab 1, while Lab 2 recruited the remaining 16 (21.9 ± 1.1 yrs, 68.9 ± 2.3 cm, 66.4 ± 3.2 kg, G1 n = 8, G2 n = 8). Subjects were required to be at least recreationally active (≥ 3 days/week of moderate to vigorous intensity exercise), free of any disease or disorder which may produce confounding results, non-smokers, and have abstained from creatine or nitrate supplementation for the month immediately prior to beginning CN supplementation, as assessed by pre-participation health history, exercise, and supplementation questionnaires.

### Measurements

All measurements were taken prior to and at the conclusion of the 28-day supplementation period. Following a 10-hour fast, all subjects submitted a blood sample for analysis in the morning to prevent diurnal variations. All blood draws were performed via venipuncture by a trained phlebotomist. Samples were analyzed for complete metabolic panels and complete blood counts by an external laboratory (Laboratory Corporation of America, Denver, CO; ANY LAB TEST NOW, Tampa, FL). All collected samples were analyzed for the following markers: white blood cell count (WBC), red blood cell count (RBC), hemoglobin, hematocrit, mean corpuscular volume (MCV), mean corpuscular hemoglobin (MCH), mean corpuscular hemoglobin concentration (MCHC), red blood cell distribution width (RDW), platelets (percent and absolute), neutrophils (percent and absolute), lymphocytes (percent and absolute), monocytes (percent and absolute), eosinophils (percent and absolute), basophils (percent and absolute), serum glucose, blood urea nitrogen (BUN), creatinine, estimated glomerular filtration rate (eGFR), BUN:creatinine, sodium, potassium, chloride, carbon dioxide, calcium, protein, albumin, globulin, albumin:globulin, bilirubin, alkaline phosphatase, aspartate aminotransferase (AST), and alanine aminotransferase (ALT). Intra-test Coefficient of Variation (CV) for Lab 2 blood measurements were all under 3%. Inter-test reliability results from 12 men and women measures up to one week apart from Lab 1 resulted in no significant differences from day-to-day (p > 0.05) and an average inter-test %CV of 6.9%.

### Statistical analyses

Pooled data was analyzed using a 3x2 repeated measures ANCOVA model for all group, time, and group by time interactions with the pre value as the covariate. A bonferroni post-hoc analysis was used to locate differences. Shapiro-Wilk tests were used to determine normality of the data. The Minimal Difference (MD) needed to be considered real was determined using the method previously described by Weir [30]. Data are presented as mean ± standard deviation. All data were analyzed using Statistica software (Statsoft, 2011).

## Results

### Pooled

Significant group by time interactions were present for absolute lymphocytes (p < 0.05). Wherein, G1 increased to a lesser extent than CRL, G2 decreased significantly relative to CRL, and G2 significantly decreased from pre to post. Absolute lymphocytes had a normal distribution at baseline (p = 0.55), yet the distribution was negatively skewed (p < 0.05) after the supplementation period. Significant group by time interactions were present for absolute monocytes (p < 0.05). G1 increased relative to CRL and G2 increased to a lesser extent than G1. Absolute monocytes was positively skewed at both time points (p < 0.05). All data is presented in Additional file 1: Table S1.

### Inter-laboratory comparisons: one serving

Significant group by time interactions were present for RDW (p < 0.05). Lab 2 observed greater increases in RDW relative to Lab 1 and significantly increased from pre to post. Significant group by time interactions were observed for platelets (p < 0.05). Lab 1 observed a significant decrease in platelets relative to CRL, and Lab 2 observed a significant increase in platelets compared to decrements in both CRL and Lab 1. Platelets were normally distributed at baseline (p = 0.17), and negatively skewed (p < 0.05) at post. Significant group by time interactions existed for absolute monocytes (p < 0.05). Wherein, Lab 2 observed significant increases compared to Lab 1 and CRL. Significant group by time interactions were present for creatinine (p < 0.05). Lab 2 observed significant decrements relative to CRL. A significant group by time interaction was present for BUN:creatinine (p < 0.05). Wherein, Lab 2 observed significant increases pre to post. A significant group by time interaction was observed for sodium (p < 0.05). Lab 2 observed a significant increase over CRL. A significant interaction was observed for serum protein (p <0.05). Lab 2 observed a significant increase relative to CRL. Significant group by time interactions were observed for ALT (p < 0.05). Lab 2 observed greater decrements relative to CRL and Lab 1. ALT was positively skewed at baseline (p < 0.05), but it was normally distributed (p = 0.52) at post. RDW, platelets, absolute monocytes, creatinine, BUN:creatinine, sodium, and serum protein were either normally distributed, negatively skewed, or positively skewed at baseline without a change in skewness at post. With the exception of BUN:creatinine, all of these markers remained within clinical reference ranges, indicating clinical insignificance. All data is presented in Additional file 2: Table S2.

### Inter-laboratory comparisons: two serving

Significant group by time interactions were present for BUN:creatinine (p < 0.05). Lab 2 observed significant increases relative to CRL and Lab 1 as well as within group differences pre to post. BUN:creatinine was positively skewed at baseline (p < 0.05), and at post, it became normally distributed (p = 0.16). Significant group by time interactions were observed for ALT (p < 0.05). Wherein, Lab 1 and Lab 2 observed greater decrements than CRL. ALT was positively skewed at baseline (p < 0.05), yet it was normally distributed at post (p = 0.93). All data is presented in Additional file 3: Table S3.

## Discussion

The results of the present study confirm our hypothesis that CN supplementation will not cause abnormal changes in hematological safety markers. When analyzing the pooled data from both laboratories, significant interactions were observed only for absolute lymphocytes and monocytes, yet these remained within the accepted physiological range and not clinically significant. Also, percent lymphocytes and monocytes were unchanged. While remaining within range, we observed unusual effects between groups. For lymphocytes, the CRL group had a greater change than G1, and for monocytes, G1 increased to a greater extent than G2. These results suggest a natural variation for these markers. Alternatively, data was collected during the winter months, and subjects may have experienced these unusual outcomes as an effect of weather. Although significant interactions were observed concerning Lab 2 in the one serving treatment, it must be mentioned that many of these differences were not observed in the two serving treatment, and the CRL sample was represented solely from Lab 1. Thus, some of the differences are not likely due to CN supplementation, and variations between subjects may be due to variations in climate between Denver, CO and Tampa, FL [31]. However, the difference in absolute monocytes may have influenced the significance observed for absolute monocytes in the pooled sample. In the analysis of both the one and two serving treatments, significant interactions were observed for ALT, yet CN supplementation appeared to lower ALT relative to control, which is not a cause for concern. When analyzing the data between laboratories, BUN:creatinine from Lab 2 increased beyond the acceptable range for the two serving treatment (Additional file 3: Table S3). This was likely due to a large, yet insignificant (p = 0.40), increase in BUN, which was also slightly outside the acceptable range after the supplementation period. However, subjects were already at the high end of the acceptable range, and the magnitude of change was attenuated when examining a larger sample (Additional file 1: Table S1).

Analysis of clinical significance at the individual level was conducted using the MD statistic that calculates the (biological and reliability) error needed to be exceeded in order for an individual measurement to be considered real as described by Weir [30]. If a subject exceeded the MD, the change was considered a true change. Variables that were significantly different at the group level were evaluated at the individual level and subjects with changes that exceeded MD were evaluated to determine clinical significance. Clinical significance at the individual level was reached when a score that exceeded the MD crossed the upper or lower limits for each variable. In the pooled analysis, one subject in G2 experienced a decrease in absolute monocytes, bringing them within the clinical reference range, and two subjects from G1 equally increased, entering the clinical reference range, and decreased, exiting the reference range. In both cases, the supplement does not appear to be causing specific, directional changes in absolute monocytes. In the 1 serving inter-laboratory comparison, 2 subjects from Lab 2 demonstrated increases in RDW, which caused them to surpass the upper limit of the clinical reference range. One subject from Lab 2 decreased, leaving the accepted range, for creatinine. One subject from Lab 2 increased, leaving the accepted range, for BUN:Cr. From Lab 1, one subject experienced a decrease in ALT, entering the accepted range. In the 2-serving comparison, 4 subjects from Lab 2 increased; 2 started in and remained outside the range, and 2 exited the range. All subjects remained within 3 standard deviations of the mean and exceeded the MD. It is also worthy to note that 8 subjects in the CRL group experienced changes in at least one variable that exceeded the MD and was outside the accepted range. Collectively, individual analyses supports our hypothesis and also supports the notion of variability. Additionally, absolute lymphocytes in the pooled analysis, platelets and ALT in the 1 serving inter-laboratory analysis, and BUN:creatinine and ALT in the 2 serving inter-laboratory analysis were distributed differently from pre to post, increasing the probability for type 1 statistical error [32].

The present findings generally agree with previous literature. The safety of creatine has been confirmed on several occasions [33], including several clinical populations [34,35]. Shelmadine et al. [36] examined the safety of a multi-ingredient supplement containing creatine, ingredients known to influence the nitrate/nitric oxide pathway, and other ingredients over an identical time period of 28 days. These researchers observed no change in clinical serum or whole blood chemistry markers, and they concluded that the supplement was safe for consumption for this duration. Other multi-ingredient supplements containing creatine have confirmed its safety for a period of up to nine weeks [37,38].

While creatine monohydrate is recognized as safe, the nitrate component of CN may still be troublesome to athletes or practitioners. Concerns over nitrate and other nitrosamines began as early as 1970. Lijinsky and Epstein [28] published a manuscript identifying dietary nitrosamines as carcinogens. However, more recent evidence suggests that dietary nitrate is safe for human consumption [39]. Moreover, dietary nitrate has been reported to reduce blood pressure [19,21,25]. Webb et al. [40] provided subjects with beetroot juice high in nitrates and observed lower systolic blood pressure after only one hour, while diastolic blood pressure and mean arterial pressure remained significantly lower after 24 hours. These findings identify nitrate as possibly beneficial to long-term health. In a review of nitrate supplementation, Hoon and colleagues [41] reported no major health consequences of nitrate supplementation, and only one minor adverse event, the discoloration of urine, which is attributed to those studies which supplemented with beet-root juice. However, provided that creatine’s safety has been so thoroughly investigated, any health complication from CN is likely due to the nitrate component.

This is the first study to examine the safety of CN. From the present results, we can conclude that CN in doses of up to 2 g are safe for human consumption for a duration of 28 days. All measured variables remained within the normal range across groups, with the exception of BUN which was not statistically significant when the groups were compared. Therefore, CN supplementation may be contraindicated for those already high in BUN. Additionally while the differences observed for absolute monocytes and lymphocytes appear to be due to variability and remained within range, CN may be unadvisable for daily consumption for those with a weakened immune system. In the present study, CN was supplemented for only 28 days, and future research may be interested in examining CN for a longer trial period to confirm its safety. Moreover, future studies are required to determine the efficacy of CN, as the combined effects of nitrates and creatine on both longitudinal and acute changes in performance and body composition are currently unknown.

## Additional files

Additional file 1: Table S1. Pooled data from Lab 1 and Lab 2. All data are presented as mean ± standard deviation. * denotes significantly different from CRL, ^a^ denotes significantly different from pre value, and ^b^ denotes significantly different from G1. Additional file 2: Table S2. Data comparison between Lab 1 and Lab 2 for the 1 serving treatment. All data are presented as mean ± standard deviation. * denotes significantly different from CRL, ^a^ denotes significantly different from pre value, and ^b^ denotes significantly different from Lab 1. Additional file 3: Table S3. Data comparison between Lab 1 and Lab 2 for the 2 serving treatment. All data are presented as mean ± standard deviation. * denotes significantly different from CRL, ^a^ denotes significantly different from pre value, and ^b^ denotes significantly different from Lab 1.

## Abbreviations

PCr: Phosphocreatine
ADP: Adenosine diphosphate
ATP: Adenosine triphosphate
CN: Creatine nitrate
Lab 1: MusclePharm Sports Science Institute
Lab 2: University of Tampa Human Performance and Sports Nutrition Laboratory
WBC: White blood cell
RBC: Red blood cell
MCV: Mean corpuscular volume
MCH: Mean corpuscular hemoglobin
MCHC: Mean corpuscular hemoglobin concentration
RDW: Red blood cell distribution width
BUN: Blood urea nitrogen
eGFR: Estimated glomerular filtration rate
AST: Aspartate aminotransferase
ALT: Alanine aminotransferase
MD: Minimal difference
